# *PLS3* Mutations in X-Linked Osteoporosis: Clinical and Genetic Features in Five New Families

**DOI:** 10.1007/s00223-023-01162-4

**Published:** 2023-12-03

**Authors:** Adriana Costa, Andreia Martins, Catarina Machado, Elena Lundberg, Ola Nilsson, Fan Wang, Alice Costantini, Symeon Tournis, Jakob Höppner, Corinna Grasemann, Outi Mäkitie

**Affiliations:** 1 Department of Pediatrics, Hospital Prof. Doutor Fernando Fonseca EPE, Amadora, Portugal; 2 Department of Pediatrics, Institution of Clinical Science, Umea University, Umeå, Sweden; 3 Division of Pediatric Endocrinology and Center for Molecular Medicine, Department of Women’s and Children’s Health, Karolinska Institutet and Karolinska University Hospital, Stockholm, Sweden; 4 Department of Medical Sciences, Örebro University and University Hospital, Örebro, Sweden; 5 Department of Molecular Medicine and Surgery and Center for Molecular Medicine, Karolinska Institutet, and Clinical Genetics, Karolinska University Hospital, Stockholm, Sweden; 6 Laboratory for Research of the Musculoskeletal System “Th. Garofalidis”, Medical School, University of Athens, Athens, Greece; 7 Department of Pediatrics, St. Josef-Hospital Bochum, Ruhr-University Bochum, Bochum, Germany; 8 Endocrine Unit, Massachusetts General Hospital and Harvard Medical School, Boston, MA USA; 9 Research Program for Clinical and Molecular Metabolism, Faculty of Medicine, University of Helsinki, Helsinki, Finland; 10 Children’s Hospital and Pediatric Research Center, University of Helsinki and Helsinki University Hospital, Helsinki, Finland; 11 Folkhälsan Research Center, Helsinki, Finland

**Keywords:** Early-onset osteoporosis, Fragility fractures, Osteoporosis in children, *PLS3*

## Abstract

Childhood-onset osteoporosis is a rare but clinically significant condition. Studies have shown pathogenic variants in more than 20 different genes as causative for childhood-onset primary osteoporosis. The X-chromosomal *PLS3*, encoding Plastin-3, is one of the more recently identified genes. In this study, we describe five new families from four different European countries with *PLS3*-related skeletal fragility. The index cases were all hemizygous males presenting with long bone and vertebral body compression fractures. All patients had low lumbar spine bone mineral density (BMD). The age at the first clinical fracture ranged from 1.5 to 13&nbsp;years old. Three of the identified *PLS3* variants were stop-gain variants and two were deletions involving either a part or all exons of the gene. In four families the variant was inherited from the mother. All heterozygous women reported here had normal BMD and no bone fractures. Four patients received bisphosphonate treatment with good results, showing a lumbar spine BMD increment and vertebral body reshaping after 10 months to 2 years of treatment. Our findings expand the genetic spectrum of *PLS3*-related osteoporosis. Our report also shows that early treatment with bisphosphonates may influence the disease course and reduce the progression of osteoporosis, highlighting the importance of early diagnosis for prompt intervention and appropriate genetic counseling.

## Introduction

Osteoporosis results in bone fragility and has been typically divided into primary and secondary forms, with osteogenesis imperfecta (OI) representing the prototypical primary osteoporosis in childhood [1, 2]. Unlike secondary osteoporosis, which is often a consequence of a chronic disease, primary childhood-onset osteoporosis is much rarer and often a monogenic disorder that disrupts the normal synthesis and turnover of bone or cartilage [3]. An understanding of the causes of osteoporosis is important for its prevention, diagnosis, and treatment.

Currently, the term early-onset osteoporosis (EOOP) refers to osteoporosis occurring in children and young adults. According to the International Society for Clinical Densitometry (ISCD) the diagnosis of EOOP in children requires a clinically significant fracture history and a bone mineral density (BMD) *z*-score at or below − 2.0 measured at the lumbar spine (LS) or total body. However, the presence of one or more spinal compression fractures in the absence of major trauma constitutes osteoporosis, even if the BMD is normal [4–6].

In premenopausal women and males < 50 years the diagnosis of EOOP is based on a low BMD, defined as a BMD *Z*‐score ≤ − 2.0 or *T*‐score ≤ − 2.5 at the LS or femoral neck, when associating with either fragility fractures or an underlying chronic illness (secondary EOOP) [5, 6]. For men aged 50 and older and postmenopausal women osteoporosis is diagnosed with a DXA BMD *T*-score below − 2.5 [6].

During the last 10 years, studies have shown pathogenic variants in more than 20 different genes that can cause childhood-onset primary osteoporosis [7]. *PLS3,* encoding plastin-3, is one of the more recently identified genes underlying childhood-onset primary osteoporosis. *PLS3* (OMIM 300131), which is located on chromosome Xq23 and has 16 exons, codes for the protein plastin-3, which is widely expressed in solid tissues and thought to be involved in cytoskeleton remodeling. Plastin-3 functions as an actin-binding protein participating in the dynamic assembly and disassembly of the actin cytoskeleton. In bone, plastin-3 has been suggested to either be part of the osteocytes’ mechanosensing apparatus or the mineralization process. Moreover, *PLS3* has an important role in the development of neuromuscular junctions and some studies are revealing a potential role in muscle fibers [3, 7–11].

Several families worldwide with variable forms of *PLS3*-related skeletal fragility have been reported. Most of these are frameshift and nonsense mutations, both likely to be followed by nonsense-mediated mRNA decay [1, 6, 7, 11]. Large intragenic deletions or duplications, leading to destroyed gene structure, and splice-site mutations, resulting in altered protein length, are also common [1, 12–16].

In general, due to *PLS3*’s location on the X chromosome, loss of function affects males more severely than females. Heterozygous female carriers have a variable phenotype from normal to low BMD and may be symptomatic even in childhood. Males with *PLS3*-related X-linked dominant osteoporosis exhibit a skeletal disorder characterized by compromised bone strength, low bone formation, and defective mineralization [13–15]. This leads to prevalent peripheral and spinal fractures and loss of adult height, usually without the characteristic extra-skeletal manifestations of OI such as joint hyperlaxity, blue sclerae, or dentinogenesis imperfecta. Recent studies indicate that defective *PLS3* function leads to severe abnormalities in vertebral morphology already in early childhood and to significant spinal pathology by early adulthood [7, 16].

The investigation of rare Mendelian disorders with decreased BMD as a key diagnostic feature constitutes a strategy for identifying genetic determinants of osteoporosis. Moreover, after gene discoveries it is important to further explore the phenotypic features and the natural course of the identified genetic entities. Since relatively few families with *PLS3* mutations have so far been described in the literature, the features and genetic variants of *PLS3*-related osteoporosis have not been fully characterized. In this report, we describe five new families with novel *PLS3* variants leading to early-onset primary osteoporosis.

## Methods

### Subjects

In this study we describe five new families with *PLS3*-linked skeletal fragility. The index cases with various *PLS3* variants associating with primary osteoporosis were identified in hospitals in four different European countries (Sweden, Greece, Germany, and Portugal).

All index subjects met the ISCD criteria for osteoporosis in children and young adults [4–6]. Secondary causes of osteoporosis were excluded by medical history, clinical examination, and appropriate laboratory analyses.

Data on patients’ clinical diagnoses, diagnostic investigations, and disease course were collected from hospital records. The information was collected separately by the team responsible for each family’s investigation. All index cases underwent clinical examination, blood sampling, skeletal radiography, bone densitometry, and genetic analyses.

The study was approved by the Research Ethics Committee of Stockholm, Sweden. A written informed consent was obtained from all participants and/or their legal representatives before reporting the data.

### Genetic Analysis

Various genetic methods were applied to identify the disease-causing gene variant in each family, according to local practices, including single-gene analysis by Sanger sequencing, gene panels using next-generation sequencing, and exome sequencing. Sanger sequencing was also used to confirm *PLS3* variants in family members.

## Results

Thanks to an international collaboration we identified altogether five families with different *PLS3* variants leading to EOOP in one or more family members. We describe the clinical features of the five index patients and the genetic variants identified in altogether ten mutation-positive individuals. The data are also summarized in Table 1.

**Table 1:** Clinical characteristics, fracture history, BMD scores at the time of first evaluation from the index patients of five families with a pathogenic variant in the gene encoding plastin-3 (*PLS3*)

Index patient	*PLS3* mutation	Inheritance pattern	Age at first fracture (years)	Height/BMI (*z*-score, SD)	Long bone fratures (*n)*	VCFs	Laboratory and other evaluations	BMD before therapy (*z*-score, SD)	BMD after therapy (*z*-score, SD)	Other clinical findings
Family 1	Hemizygous frameshift variant c.1543del (p.Asp515Metfs*11)	XL	2	+ 2/+ 1	8 (Ulna, radius, humerus, tibia, and metacarpals)	T5 C3–C5	Dental examination noted poor trabecular bone in the jaw bone, but was otherwise normal General endocrine/metabolic laboratory and ophthalmology assessments have been unremarkable	LS − 1.6 (0.538 g/cm^2^) Total body less head − 0.9 (0.548 g/cm^2^)	? (Pamidronate)	Pectus excavatum, flat feet, broad and short thumbs and digit 4–5 syndactyly
Family 2	Hemizygous nonsense variant c.827G>A (pTrp276*)	XL	13	− 2/− O.5	2 (radius)	L1	Full laboratory investigations were unremarkable	LS − 3.7 (0.748 g/cm^2^) FN − 2.9 (0.665 g/cm^2^) TH − 3.0 (0.678 g/cm^2^) Radius ultradistal − 5.0 (0.274 g/cm^2^)	FN − 2.8 (0.681 g/cm^2^) TH − 2.7 (0.706 g/cm^2^) (10 months teriparatide) LS − 4.9 (0.612 g/cm^2^) (15 months teriparatide)	–
Family 3	Hemizygous nonsense variant c.994_995delGA (p.Asp332*)	De novo variant	5	− 1/− 1	2 (Tibia, femur)	Multiple vertebral bodies	Laboratory parameters of bone metabolism were repeatedly within age-appropriate norms	LS − 2.9	LS − 2.6 (4 months pamidronate)	Pectus carinatum, Growth hormone deficiency
Family 4	Hemizygous deletion X:114771600-114890428	XL	1.5	− 1/− 2	7 (Forearm, wrist, elbow, and clavicle)	L2	Increased alkaline phosphatase and slightly increased PTH (67 ng/l; normal 15–65 ng/l) with vitamin D deficiency (18.4 μg/l; normal 30–70 μg/l)	LS − 3	LS − 2.67 (2 years pamidronate)	Pectus carinatum, joint hypermobility
Family 5	Hemizygous deletion X:114644041-115418576	XL	8	0/+ 2	0	From C7 to L5, it spares D1, D2, D10, and L1	Bone resorption marker type 1 collagen N telopeptides in 24-h urine were greatly increased (1194 nM BCE/mM creatinine). General, endocrine/metabolic laboratory investigation were unremarkable	LS − 5 (0.299 g/cm^2^) FN − 5.6 (0.406 g/cm^2^) TH − 6.7 (0.320 g/cm^2^)	LS + 0.7 FN + 0.3 TH − 4.1 (1 year pamidronate)	–

All index patients are males. According to ISCD criteria, only clinically significant fracture history was considered (minor fractures, such as fingers, were excluded). The reference sequence for the variants described in families 1–3 is NM_005032.6

*BMI* body mass index, *BMD* bone mineral density, *FN* femoral neck, *LS* lumbar spine, *TH* total hip, *VCF* vertebral compression fractures, *XL* X-linked

### Family 1

The proband is an 8-year-old Swedish boy referred for investigation due to multiple low-energy fractures. At 2 years of age, he had his first fracture after minimal trauma. By 8 years, he had had eight fractures after minor trauma (ulna, radius, humerus, tibia, and metacarpals).

**Fig. 1:**
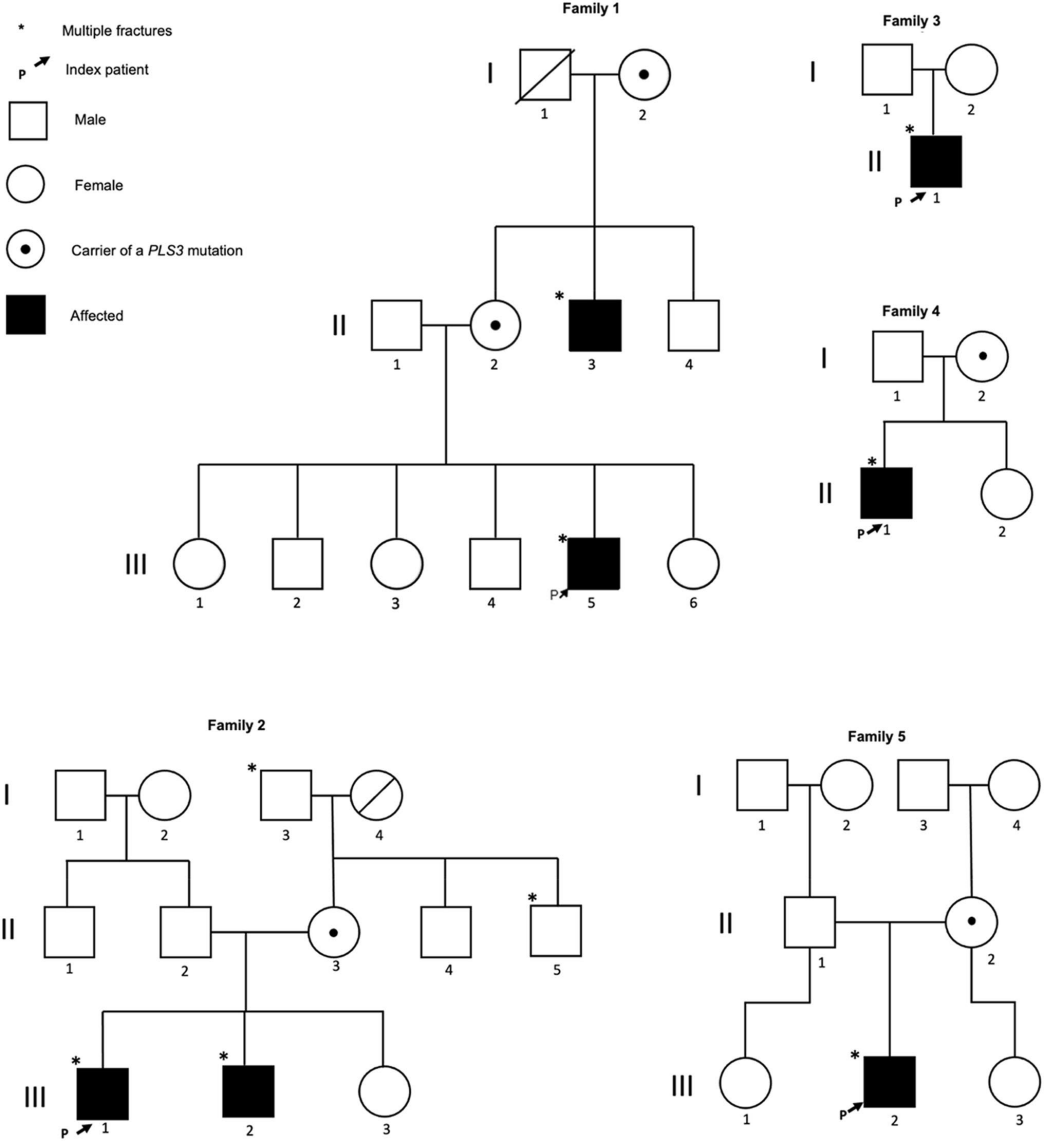
Families pedigrees: Pedigrees of the five families presented with mutation of the *PLS3* gene

He is the fifth out of six children of healthy European parents and had an unremarkable neonatal and childhood period. None of the parents have a history of skeletal disease. The patient’s maternal uncle, age 38 years, has a history of multiple fractures and severe back pain requiring analgesic medication since young age (Fig. 1), having a clinical diagnosis of Osteoporosis pseudoglioma syndrome since 11&nbsp;years old.

On physical examination at 8 years, the proband’s height was 142.5 cm (+ 2.0 SD) and body mass index (BMI) 17.9 kg/m^2^ (+ 1.0 SD). He had pectus excavatum, flat feet, broad and short thumbs and digits 4–5 syndactyly bilaterally. Dental examination with radiography showed poor trabecular bone in the jaw bone, but was otherwise normal.

**Fig. 2:**
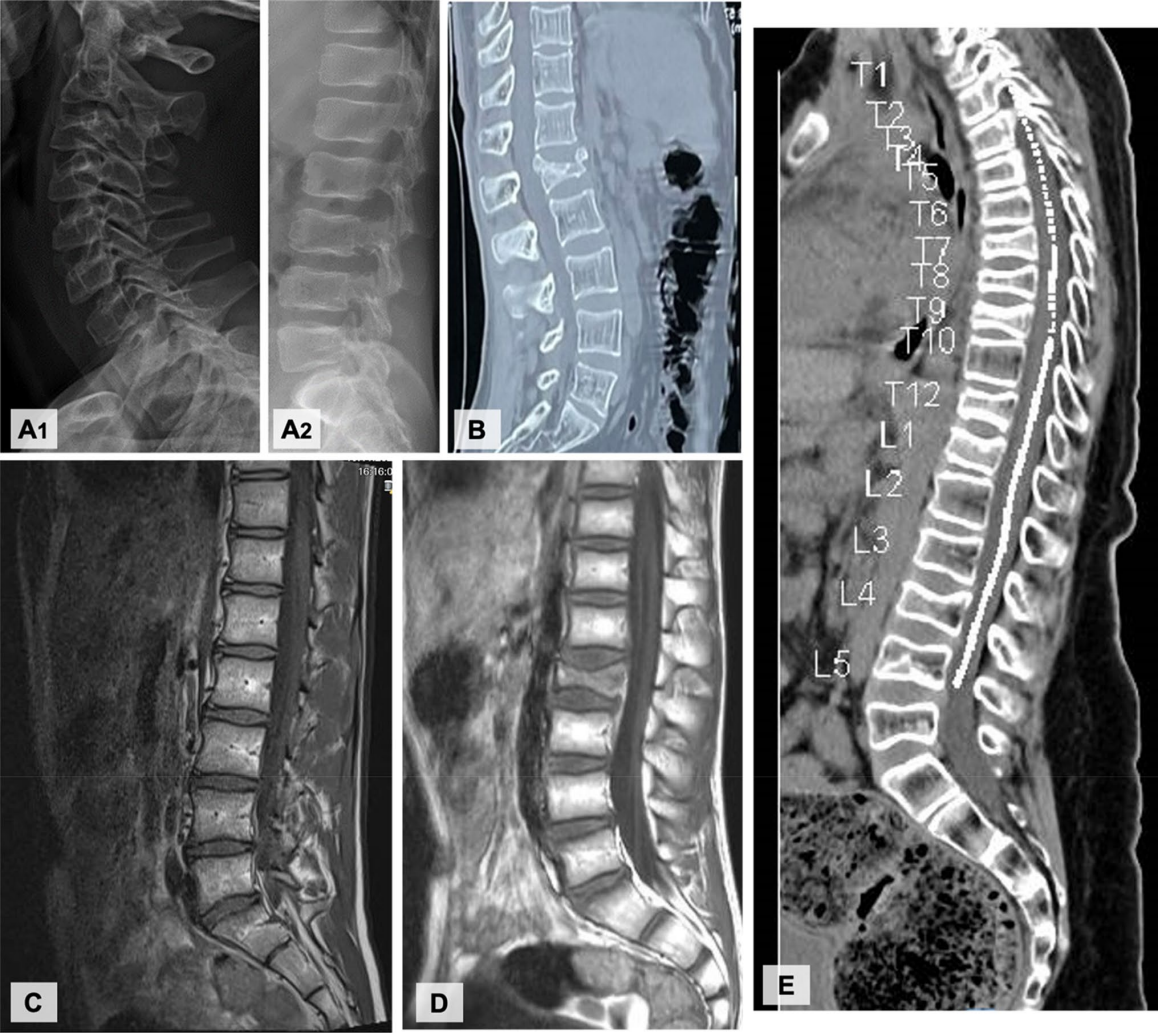
Spinal images: collapse and a reduction of height of multiple vertebral bodies: **A** Family 1 index patient’s x-ray; **B** Family 2 index patient’s CT; **C** Family 3 index patient’s MRI; **D** Family 4 index patient’s MRI; **E** Family 5 index patient’s MRI

The BMD measurements showed a *Z*-score of − 1.6 at lumbar spine (LS). Spine x-ray showed reduced height (approximately 40%) of T5 and slightly wedged vertebral bodies C3–C5 (Fig. 2A).

**Fig. 3:**
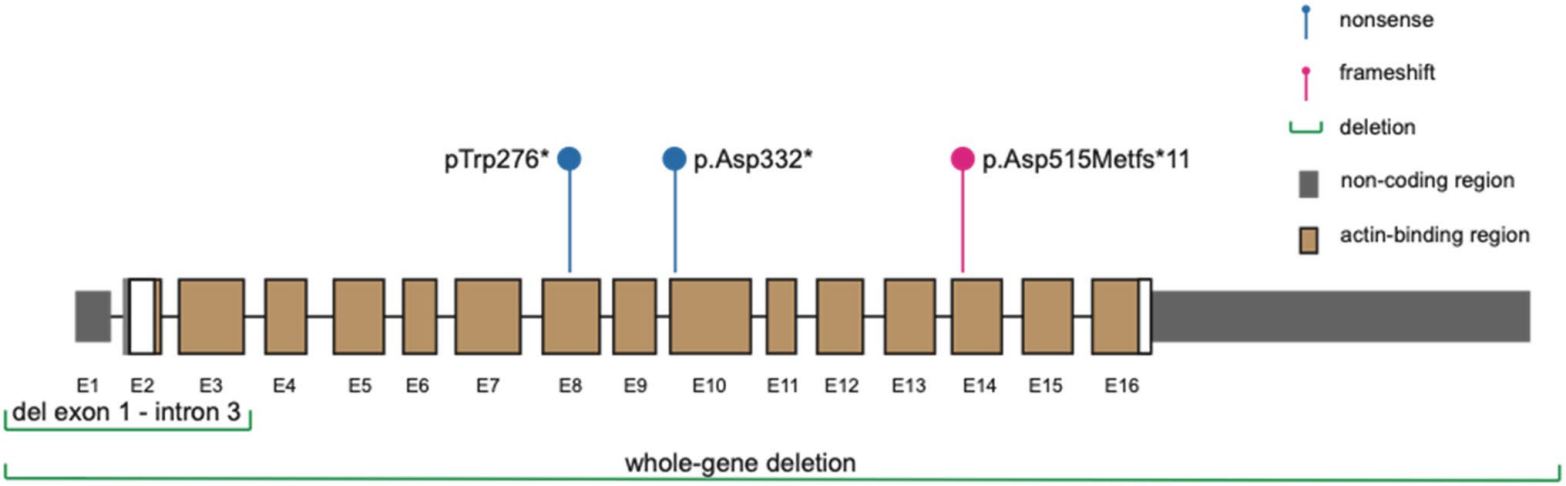
*PLS3* variants: Schematic representation of *PLS3* the RNA-splicing form NM_005032.6 showing the location of the 5 identified variants. E1-16 = exons 1–16

Genetic analysis with a gene panel (Blueprint Genetics) identified a hemizygous frameshift variant in *PLS3* [c.1543del (p.Asp515Metfs*11)] in the proband (Fig. 3). This variant was classified as likely pathogenic and was inherited from the unaffected mother who also harbored the same *PLS3* variant. This variant is predicted to cause loss of normal protein function through protein truncation and/or nonsense-mediated mRNA decay. The variant is absent in gnomAD and has not been described in the medical literature or reported in any disease-related variant database. The DNA sample of the mother’s brother, who also had osteoporosis, was not available for testing.

The patient has been treated with bisphosphonates (pamidronate monthly) for the last 10 months and has not experienced any new fractures during this period.

### Family 2

This 26-year-old European man was referred due to unstable fracture at L1 vertebra after a low impact trauma, managed with posterior spinal fusion (Fig. 2B). He had a past history of radius fractures, at the age of 13 and 15 years, following sports injury. The physical examination was unremarkable (height 162 cm − 2.0 SD, BMI 23 kg/m^2^ + 0.5 SD).

He was born from healthy non-consanguineous parents. There was a family history of multiple fractures in his grandfather and his uncle, from his mother’s family arm (Fig. 1).

The BMD *Z*-score (GE, Lunar Prodigy Pro) at the LS was − 3.7. BMD measurements of the family members revealed that his brother, aged 20 years, had low BMD at LS (*Z*-score − 3.1), FN (*Z*-score − 2.4), and Radius 33% (*Z*-score − 3.0). His father had osteopenia at LS (*T*-score − 2.3) and FN (*T*-score − 1.5). His sister aged 21 years and his mother aged 46 years had normal BMD, LS *Z*-score − 0.8 and − 1.7, respectively.

Sanger sequencing revealed a novel nonsense variant in *PLS3*, c.827G>A (pTrp276T*), in the index case (RefSeq NM_005032) (Fig. 3). This variant was maternally inherited and the index’s brother also harbored the same variant.

The proband was started on teriparatide treatment along with calcium and vitamin D. Ten months after teriparatide, BMD *Z*-score of the FN was − 2.8 and TH − 2.7, increased by 2.4% and 4.1%, respectively, while radius BMD did not change. BMD at LS (0.579 g/cm^2^/ *Z*-score − 5.1) decreased by 22%. No fractures or adverse events occurred during the 10-month follow-up. One possible explanation for the substantial decline at the LS (as opposed to the improvement at the hip) is the trajectory of BMD following spinal fusion surgery. Indeed, 5 months later, BMD at the LS increased by 5.7% (*Z*-score − 4.9).

### Family 3

The patient is a now 20-year-old man, the only child of a healthy non-consanguineous couple of Russian descent. The parents are of average height without a fracture history (Fig. 1). He presented to pediatric endocrinology at the age of 7 years for evaluation of short stature. At that time, he had suffered a finger fracture after a fall, at the age of 5 years, followed by a fracture of the left femur and of the right tibia due to minor falls. A diagnosis of growth hormone (GH) deficiency was established based on insufficient GH levels in two GH stimulation tests at 9 years of age and he was started on recombinant human GH. GH treatment was discontinued at 17 years when the annual growth rate fell below 3 cm. The final height is 176 cm (− 0.6 SD), which falls in the range of the parental target height and he developed a mild pectus carinatum.

BMD by DXA at 9 years revealed a *Z*-score of − 2.8 at the LS. Following the initiation of GH therapy, the *Z*-score improved to − 2.5 at age 13, but at 16 years, the *Z*-score had dropped to − 2.9. Magnetic resonance imaging (MRI) revealed collapse of multiple vertebral bodies (Fig. 2C).

Genetic testing showed an X-linked inherited osteoporosis caused by a hemizygous mutation in the *PLS3* gene [c.994_995delGA (p.Asp332*)]. This diagnosis was established at the age of 18 years (Fig. 3).

Treatment was started with vitamin D and calcium supplementation and intravenous bisphosphonate (pamidronate). In the 1st year he only received 3 mg/kg body weight of pamidronate. It was intended to administer 9 mg/kg BW for the 1st year. However, due to the onset of the coronavirus disease pandemic in 2020 the follow up visits had to be canceled. Treatment was switched to zoledronate and he received two doses of 4 mg in 6 months’ intervals. At 18 years (after the low dose of pamidronate), the LS BMD had mildly improved with a *Z*-score − 2.6. At the same time an X-ray of the spine showed normal height of the vertebrae of the thoracic and lumbar spine. During follow-up no fractures occurred.

### Family 4

The index patient is a currently 17-year-old adolescent of Polish descent treated for X-linked juvenile osteoporosis and a pectus carinatum in the endocrine and bone health clinic of a tertiary university hospital. His height is 170.7 cm (− 1 SD) and the BMI is 17.8 kg/m^2^ (− 2 SD).

The first fracture occurred at the age of 1.5 years and by 8 years of age, six additional fractures (forearm, wrist, elbow -twice- and clavicle) had occurred. At 13 years of age a fall resulted in a vertebral fracture of L2, which required spinal fusion surgery (Fig. 2D) and he was presented to a specialized bone clinic for suspected OI. Physical examination revealed marked hypermobility of the joints and a mild thoracic asymmetry.

Family history revealed back pain in the mother, but fractures had never occurred. The patient's maternal grandfather had suffered from fractures sporadically, but due to adequate trauma. The patient has a healthy sister (Fig. 1).

A DXA scan at 12 years revealed a LS *Z*-score of − 2.9. A follow up at age 13.5 years showed a *Z*-score of − 3.0 after the osteosynthesis material was removed. Molecular genetic testing revealed a hemizygous deletion starting before the *PLS3* gene and spanning into intron 3 of the same gene (chromosomal coordinates X:114771600-114890428) (Fig. 3). Genetic testing of the mother revealed that she is a heterozygous carrier of the deletion (DXA with LS *T*-score of − 1).

Due to the multiple fractures and reported back pain an intravenous therapy with pamidronate (9 mg/kg BW in the 1st year, 5 mg/kg BW subsequently) was started at the age of 13.5 years. A thoracic x-ray at age 15.5 years showed a loss of height at thoracic vertebra Τ12. The corresponding DXA measurement revealed a LS *Z*-score of − 3.5 (− 2.7 after adjusting for height). Even though the DXA *Z*-score did not improve with the therapy, there have been no further fractures since the treatment with bisphosphonates was started.

### Family 5

An 11-year-old Portuguese boy, the only child of a healthy Portuguese couple (Fig. 1), has a past history of two finger fractures after a sports injury at the age of 8 years and obesity. At 11 years-old, he was 144.9 cm tall (− 0.07 SD) with a weight of 60.9 kg (+ 2 SD) and BMI of 29 kg/m^2^ (+ 2 SD). He was admitted to the hospital due to a 3-month progressive worsening of lumbar pain with increasing difficulty to walk due to multiple spinal fractures. There was no history of trauma. The physical examination was unremarkable. His 36-year-old father had lumbar spine osteoporosis but no fractures. The mother is healthy, has no history of bone fractures and has a normal BMD.

An MRI of the spine revealed multiple vertebral body fractures (from C7 to L5, only sparing T1, T2, T10, and L1) with collapse and a height reduction of 25–50% (Fig. 2E). Full body X-ray scan revealed diffuse osteopenia but did not find any other fractures. The DXA revealed a *Z*-score of − 5.0 in LS. Neurological, cardiological, ENT, and ophthalmological evaluations were normal.

Next-generation sequencing (Illumina) of genomic DNA for 26 genes, including *PLS3*, was performed (Human All Exon V6, Agilent Technologies) and led to the identification of a novel hemizygous deletion removing the whole *PLS3* gene (Fig. 3). Array comparative genomic hybridization, CytoScan 750 K, confirmed the presence of a 775 kb deletion on chromosome X (coordinates X:114644041-115418576) removing the entire *PLS3* gene as well as AGTR2, encoding type-2 angiotensin II receptor.

A heterozygous variant of unknown significance (VUS) in *LRP5* was also identified [NM_002335.3:c.3443C>T(p.Thr1148Ile)]. The patient inherited the *PLS3* deletion from his unaffected mother and the *LRP5* variant from his father with spinal osteoporosis but no fractures.

The patient started quarterly intravenous pamidronate cycles (1 mg/kg/day for 3 days), calcium and vitamin D. After 1 year of treatment the DXA revealed a general increase in all BMD values (*Z*-score + 0.7 in LS) and regression of all symptoms, with no new fractures.

## Discussion

Several *PLS3* variants have been reported to date [1–3, 13] as summarized in Table 2. In this article, we identified a total of five pathogenic *PLS3* variants in five families with osteoporosis and osteoporotic fractures manifesting already in childhood. Due to *PLS3*’s location on the X chromosome, *PLS3* loss-of-function variants affect males more than female. Of the 28 previously reported index patients (Table 2), only one was a woman. The phenotype in affected hemizygous males was comparable in all reported patients in included multiple peripheral and vertebral body fractures.

**Table 2:** Review of genetic and clinical presentation of *PLS3* linked early-onset osteoporosis cases reported in literature

Number of family and References	*PLS3* mutation	Age at first fracture/age at study time (years)	Long bone fractures (*n*)	VCFs	BMD before therapy (*z*-score, SD)	BMD after therapy (*z*-score, SD)	Other clinical findings
1 Dijk et al. [1] (6 patients were identified in this family)	Single deleterious hemizygous frameshift c.235delT;p.(Tyr79Ilefs*6), in exon 3	2/41	13	Almost all vertebrae	LS − 5.5, FN − 3.4	LS − 4.6, FN − 3.1 (2 years bisphosphonate)	None
2 Dijk et al. [1] (2 male patients were identified in this family)	Nonsense mutation, c.1471C → T;p.(Gln491*), in exon 13	?/34	13	Multiple	LS − 3.4/FN − 3.4	–	None
3 Dijk et al. [1]	c.321T → A variant in exon 4	?/13	6	–	LS − 1.7, FN − 3.2, TB − 3.7	–	Split-hand/split-foot malformation type 3 (10q24 duplication); joint hyperlaxity; hearing loss; severe osteoarthritis of mandibular condyles
4 Dijk et al. [1]	Splice-site mutation c.748+1G>A in exon 7	–	–	Yes	–	–	None
5 Dijk et al. [1]	Insertion mutation c.759_760 insAAT in exon 8	–	Yes	Yes	LS − 2.5	–	None
6 Dijk et al. [1]	Frameshift PLS3 mutation c.1647delC in exon 15	–	Yes	Yes	LS − 2.8	–	None
7 Fahiminiya et al. [17] (2 brothers)	Hemizygous frameshift deletion in exon 10 (c.994_995delGA)	2–2/7–3	4/1	Multiple thoracic	LS − 3.5/− 1.7	LS − 0.8/LS + 0.03 (6 years alendronate)	Brother 1: clumsy gait and mild spastic cerebral palsy
8 Fahiminiya et al. [17] (2 brothers)	Hemizygous missense variant in exon 13 (c.1433T>C)	5–4/6–6	2/1	Multiple thoracic	LS − 3.4/− LS 3.3	Refuse treatment	None
9 Laine et al. [15] (2 brothers)	Hemizygous ;splice site mutation (c.73-24T>A)	8–8/12–9	4/2	Several thoracic	LS − 2.7, TH − 2/LS − 3.1, TH − 1.8	–	None
10 Nishi et al. [18] (2 brothers)	Missense mutation c. 1103C>A in exon 10 (Heterozygous missense variants also in OTOG and USH2A)	6/11	Recurrent fractures/1	–	TB − 5.6/− 4.2	–	Developmental delay; deafness; inguinal/umbilical hernia; facial dysmorphisms; blue sclerae; small joint laxity
11 Kämpe et al. [7]	Hemizygous nonsense variant (c.766C>T; pArg256*) in exon 8	10/30	6	Multiple	18 ;years old: LS − 4.1, FN − 3.3	LS − 3.8, FN − 2.8 (1-year zoledronic acid)	Blue sclerae; yellow teeth and loss of enamel; joint hyperlaxity; soft skin; minor aortic valve regurgitation; asthma
12 Kämpe et al. [7]	Missense variant in exon 12 (c.1424A>G; p.N446S)	6/10	3	3	LS − 6.6, TB − 3.5	No more fractures after start treatment. Increase BMD (1 year pamidronate + zoledronic acid 6/6 months)	Joint hyperlaxity
13 Kämpe et al. [13] (2 brothers)	Deletion of exons 4–16	11–7/11–7	0	Multiple in lumbar and thoracic spine	LS − 3.4/LS − 3.4	–	Brother 1: waddling gait (muscular hypotonia) and facial dysmorphism Brother 2: waddling gait (muscular hypotonia); facial dysmorphism; small joint laxity; opalescent teeth
14 Kämpe et al. [13]	Deletion of the entire gene—the span of the deletion was determined to be ~ 600 kb, affecting two protein-coding genes, *PLS3* and *AGTR2* and two non-coding RNA genes *DANT2* and *PLS3-AS1*	4/12	7	Multiple (all spine)	LS − 3.6	LS − 0.2, FN − 1.9, and TB − 1.4 (After bisphosphonates—time?)	None
15 Lv et al. [19]	Deletion exon10-exon16 (deletion from intron 9 to 3′UTR)	2/10	Multiple	Multiple thoracic and lumbar	LS − 3, FN − 3.4	LS 2.1, FN − 0.4 (2 years zoledronic acid)	Kyphosis
16 Kannu et al. [3]	3.411-MB deletion in chromosome region Xq23 (112,419,139–115,830,286) involved 17 RefSeq genes including *PLS3* gene	4/4	4	T4, T9, T12, L2	LS − 2.1	LS − 0.1 (2 years pamidronate)	None
17 Kannu et al. [3]	c.1730dup mutation	2/2	2	T7, L1	LS − 4	LS − 2 (2 years pamidronate)	–
18 Costantini et al. [20] (2 patients in the family)	Duplication mutation g.114,848,381_114,860,880dup	?/21	10 metatarsal fractures	Multiple	LS − 3.1	Improvement in vertebral shape (2 years bisphosphonate)	–
19 Costantini et al. [20]	Frameshift c.1096_1100delAACTT in exon 10	2.5/8	1	T5, T6, and T8	LS − 3.5, TB − 2.2	LS − 2.3, TB − 0.8 (1 year alendronate)	Blue sclerae; joint hyperlaxity; kyphosis; facial dysmorphism (epicanthic folds, narrow external ear canals, micrognathia, and a high-arched palate)
20 Collet et al. [21]	Frameshift c.1206dup in exon 11	13/?	Yes	Yes	LS − 2.3	–	–
21 Collet et al. [21]	Missense c.1876G>A in exon 18	18/?	Yes	Yes	LS − 3.9	–	–
22 Balasubramania et al. [22]	Hemizygous frameshift mutation deletion, c.1765del in exon 16	13/40	?	Almost all lower thoracic and lumbar vertebrae	LS − 4.8, TH − 3.6	–	None
23 Balasubramania et al. [22]	Hemizygous nonsense mutation c.1295T>A in exon 12	2/12	3	7 vertebral lumbar-thoracic	LS − 2.7, TB − 2.6	LS − 1.4, TB − 1.7 (6 months pamidronate) LS + 1.4 (2.5 years Pamidronate/Zoledronic acid)	None
24 Chen et al. [23]	Nonsense mutation in exon 7 (c.745G>T)	6/11	2	Multiple thoracic vertebral compression	LS − 1.2, FN − 2.1	–	Blue sclera
25 Cao et al. [24]	Splice-site mutation c.892-1G>A in intron 8	4/11	5	0	LS − 1.8, FN − 2.3	–	None
26 Hu et al. [9] (2 brothers)	Frameshift mutation c.1106_1107insGAAA in exon 10	4/12	1	Multiple vertebral	LS − 2, FN − 3.2	LS + 1, FN + 1 (24 months bisphosphonate)	Blue sclera and kyphosis
27 Wang et al. [2] (3 family members affected)	Nonsense mutation c.244C>T in exon 4	7/14	Yes	No	LS − 2.6, TH − 2.1, TB − 2.3	–	None
28 Wu et al. [12] (2 brothers)	Hemizygous splicing mutation, c.892-2A>G in intron 8	4–4/5–16	3	Multiple lumbar	LS − 3.3, FN − 4.5, TH − 4.5/LS − 1.3, FN − 3.2, TH − 2.4	Brother 1: LS − 3 (6-month bisphosphonate)	Scoliosis

Data are presented considering the index patient of each family. All index patients were males, except the proband in family 12

*BMD* bone mineral density, *FN* femoral neck, *LS* lumbar spine, *TB* total body, *TH* total hip, *VCF* vertebral compression fractures

Our report expands the genetic spectrum of PLS3-related osteoporosis. Four of the variants in the present study correspond to novel *PLS3* mutations in four different families with skeletal abnormalities. Additionally, the hemizygous nonsense *PLS3* variant c.994_995delGA (p.Asp332*) identified in one of the families was previously described in case reports in familiar cases of osteoporosis related to *PLS3* mutations [17]. The index cases were all hemizygous males presenting with vertebral body compression fractures. Only one index patient (family number 5) did not present long bone fractures. The phenotype of these patients was comparable to what has been reported in other studies [8, 9, 25]. All patients had low LS BMD and the age at the first clinical fracture ranged from 1.5 to 13 years, as described in Table 1.

The index patient in family 5 at the age of 8 years had the most severe phenotype showing compression fractures of almost all vertebral bodies with a LS *Z*-score of − 5. In this case, variants were identified in two different genes, *LRP5* and *PLS3*. A possibility of digenic inheritance should be considered and more studies, namely in other family members, need to be performed to determine the significance of the *LRP5* variant, which was classified as a variant of unknown significance.

All heterozygous women reported here had normal bone density and no bone fractures. However, other studies have reported symptomatic osteoporosis and significant spinal compression fractures also in women with heterozygous *PLS3* variants [16]. Occasionally, heterozygous females may present with more severe childhood-onset osteoporosis [7]. Therefore, it seems important that females with heterozygous *PLS3* variants are detected early in order to carry out an adequate follow up and early medical intervention.

Regarding treatment, four patients were treated with pamidronate and zoledronate and only one received teriparatide. These differences reflect different clinical practices and experiences, but also the fact that teriparatide is not used to treat pediatric osteoporosis.

Considering the increase of BMD after 15 months of teriparatide treatment in the index patient of family 2, we may conclude that *PLS3* mutation-related osteoporosis can respond to teriparatide treatment. This is in line with what was shown by Valimaki et al. who demonstrated that after 24-month treatment with teriparatide, all *PLS3* mutation-positive patients showed a minor increase in BMD without new clinical fractures [26]. Nevertheless, treatment results are variable, and only very little evidence exists. More studies and long-term follow-up are needed.

Early treatment with bisphosphonates may also positively influence the course of the diseases, since no fractures occurred once patients were started on pamidronate/zoledronate, although the BMD improved only slightly in most cases. Furthermore, as long as vertebral reshaping is partially explained by stabilization of BMD, we may hypothesize that this treatment might be an important factor in vertebral body reshaping in growing patients, as it was possible to see in vertebra imaging progress after treatment in the index patient of family three. A more complete study with a long-term follow-up would be important to understand this phenomenon. More studies are needed as well as long-term evaluation, in order to understand the efficiency of bisphosphonates treatment in *PLS3* osteoporosis patients [8].

Although these observations are in line with the previous reports [11], data still remain scarce and require further studies on the molecular mechanisms leading to severely compromised bone tissue properties in *PLS3* mutation carriers. Importantly, our study shows that a genetic diagnosis in the index case often prompted additional investigations in the family, leading to the identification of additional affected relatives. This underscores the importance of timely genetic diagnosis and extensive family history.

In conclusion, hemizygous mutations in *PLS3* cause a monogenic form of X-linked osteoporosis. Affected males often have bone pain and a history of multiple low-impact long bone and vertebral fractures, as well as thorax deformities at diagnosis. Early diagnosis is of utmost importance to prevent fractures, deformities, pain and disability, and requires a careful family history, clinical and radiological evaluation, and timely genetic testing.

Our findings expand the genetic spectrum of *PLS3*-related osteoporosis and highlight the importance of early diagnosis and early intervention with bisphosphonates for the affected individuals.
